# Early sports specialization in Japanese young soccer players and related factors

**DOI:** 10.1371/journal.pone.0302339

**Published:** 2024-08-29

**Authors:** Yasuharu Nagano, Shogo Sasaki, Ayako Higashihara, Takashi Oyama

**Affiliations:** 1 Department of Sports and Health Science, Japan Women’s College of Physical Education, Setagaya, Tokyo, Japan; 2 Department of Competitive Sports, School of Physical Education, Tokai University, Hiratsuka, Kanagawa, Japan; 3 Institute of Physical Education, Keio University, Yokohama, Kanagawa, Japan; 4 Faculty of Economics, Teikyo University, Hachioji, Tokyo, Japan; University of Naples Federico II: Universita degli Studi di Napoli Federico II, ITALY

## Abstract

Although understanding the status of sports participation is essential for preventing injuries in young athletes, the level of specialization and relevant information in Japan is unknown. This study aimed to clarify the status of sports specialization and examine the relationships between specialization and training status in Japanese young soccer players. Four hundred Japanese young male soccer players were included. The players’ parents completed a web questionnaire that consisted of three-point questions regarding specialization and training status (year, days of playing soccer, age when starting soccer). The level of specialization and accompanying information were calculated, and data were compared by specialization status. Of the participants, 53.8% demonstrated a high level of specialization. In addition, 74.5% considered soccer more important than other sports, 89.0% trained in soccer for more than 8 months of the year, and 74.0% had quit other sports to focus on soccer or played only soccer. The proportion of participants who played only soccer was significantly higher in the high-specialization group (37.6%) than in the moderate-specialization (22.5%; *P* < .01) and low-specialization (7.1%; *P* < .01) groups. By specialization status at grades 4 to 6 (9–12 years), 40.3% of participants demonstrated a high level of specialization. Young Japanese soccer players tend toward early specialization. Factors contributing to the high specialization level are being active throughout the year and rarely playing other sports. Training volume should be controlled, and an environment allowing young soccer players to participate in other sports simultaneously is needed, with early specialization being avoided.

## Introduction

For children, soccer is efficacious in improving physical capacity, health-related fitness parameters, and self-esteem [1]. Soccer has been a highly popular sport for young athletes. Meanwhile, early specialization in a particular sport and increased participation in organized sports with increased training volume have become prevalent for improving athletic performance and achieving social success. However, a previous meta-analysis found that sport specialization in athletes younger than 18 years was associated with an increased risk of musculoskeletal overuse injuries [2]. Therefore, position statements from sports medicine organizations have generally been consistent in recommending against early sport specialization [3]. To provide fundamental information for injury prevention in young athletes, specialization status must be clarified.

Sports specialization is defined as “intense, year-round training in a single sport at the exclusion of other sports” [3, 4]. To evaluate the degree of sports specialization, the three-point Jayanthi scale [3, 4] is popularly used and is based on three key components: year-round training, choosing a main sport, and quitting other sports. Previous studies have reported on the specialization status of young athletes. Post et al [5] reported that among all youth athletes (mean age 14.2 years), 41.8% had high-specialization status, and this proportion was 35.3% among soccer players. Post et al [6] also reported that the status of high sports specialization by age in young athletes including soccer as follows: 30.2% at 12 years of age, 38.1% at 13 years of age, and 42.3% at 14 years of age. Bell et al [7] reported that the proportion of high-specialization status of youth male athletes including soccer (mean age 15.7 years) was 36.9%. However, there have been no reports of the sports specialization status of Japanese young athletes.

One reason for specialization is the status of other sports played. Although a previous study showed a high percentage of young students (77.9%) played multiple sports [7], in Japan, the proportion of children who play multiple sports is very low, ranging from 10.0% [8] to 17.4% [9] for children in grades 7–9 and 22.9% for soccer players [9]. In addition, some athletes have only ever played a single sport, and athletes who continued a single specific sport from elementary school age are more likely to have overuse disorders, especially in team sports [10]. Consequently, it is possible that young Japanese athletes are highly specialized due to a low percentage of multiple sports and that their specialization is increasing as they have no or little athletic experience other than soccer. However, previous studies [8, 9] were retrospective and did not depict the current situation of young soccer players. Like in other countries, the choice of sports among Japanese youth is diverse; therefore, it is important to clarify the status of sports other than soccer among young players and examine their relationship with specialization to consider the long-term development of modern soccer players.

Another factor related to the level of specialization is training volume. In young soccer players, training volume of >5 days per week playing soccer was related to overuse injuries [5]. In some recent studies in Japan, the number of days of weekly activity was related to acute injuries and overuse injuries during grade 7–9 [9]. In addition, the number of training days per week was related to growth-related knee and heel pain in Japanese junior soccer players [11]. Other studies have focused on the number of months of sports per year, with more months spent playing sports being associated with acute injuries and overuse injuries [5]. Although it is assumed that training volume increases with the level of specialization, the relationship between training volume and the level of specialization in current young Japanese soccer players remains unclear.

Although understanding the status of sports participation is essential to prevent injuries in young athletes, the level of specialization and relevant information in Japan remains unknown. The purpose of this study was to clarify the status of sports specialization and examine the relationships between specialization and training status (only sports carrier of soccer, days spent playing soccer, and age when starting soccer) among Japanese young soccer players. Our first hypothesis is that the specialization status of Japanese young soccer players is higher than that reported in previous studies and related to only sports carrier of soccer. Our second hypothesis is that high sports specialization of Japanese young soccer players is related to more days per week of playing soccer. By clarifying the state of specialization among young soccer players in Japan, it will be possible to compare the situation with that in other countries. This will also provide a basis for considering the necessary solutions for soccer players who are becoming more specialized at an earlier age.

## Materials and methods

### Study setting

**Fig 1:**
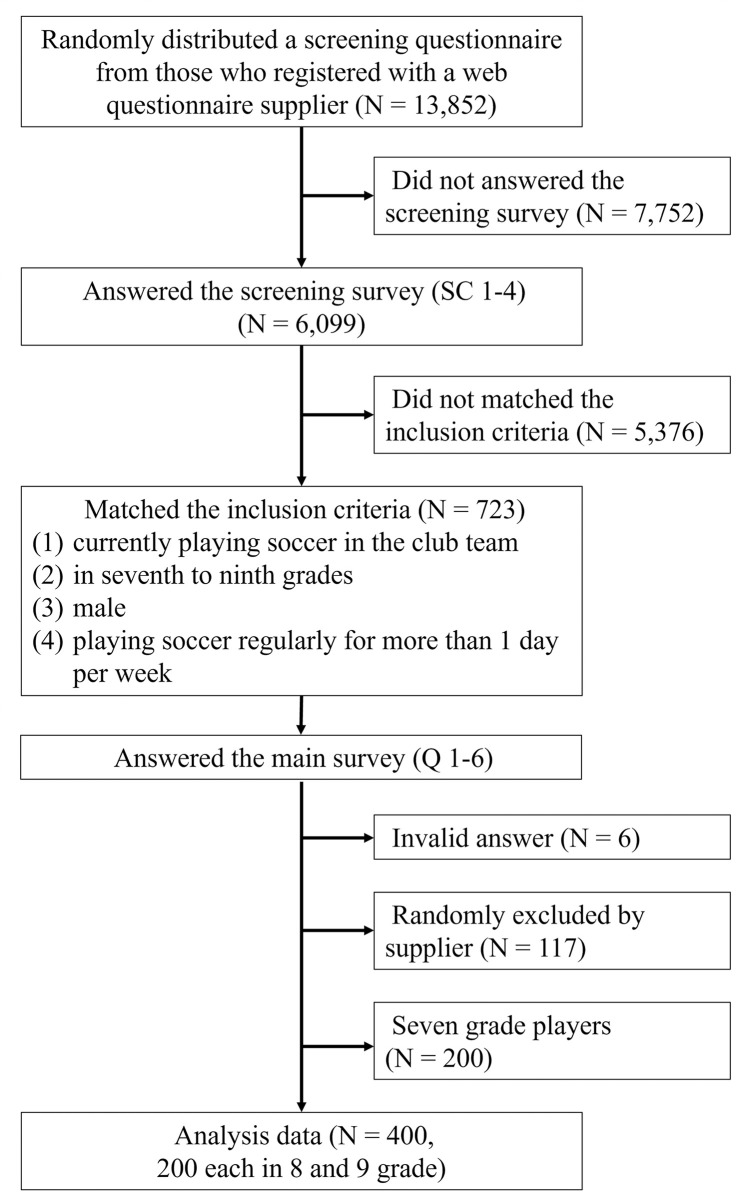
Participant recruitment flow chart.

This study was a cross-sectional study of data obtained from a web-based questionnaire and was conducted as part of a previous study [12]. The participant recruitment flow chart is shown in Fig 1. We aimed to recruit 400 subjects whose male child played soccer and attended school in grades 8 (13–14 years) or 9 (14–15 years). According to the power analysis [13], the required sample size for a descriptive study of a dichotomous variable (*P* = .40, W = 0.10, confidence level = 95%) was 369. Based on this value, we targeted a sample size of 400. Our plan was to recruit an equal number of participants from grades 8 and 9. Initially, we randomly distributed a screening questionnaire to 13,852 individuals registered with a web questionnaire supplier (Rakuten Insight Inc.), known for efficient population sampling in academic research. A total of 6,099 of the 13,852 individuals responded to the screening survey (Table 1, SC 1–4). Next, based on the results of the screening survey, 723 parents of young male soccer players in the seventh to ninth grades participated in the main survey (Table 1, Q1–6). Finally, 6 data points were excluded through data cleaning, and 600 out of the 717 collected datasets, with 200 from each school grade, were randomly selected by a questionnaire supplier. A series of surveys achieved the desired sample size in 3 days (August 1–3, 2022). Although parents of seventh-grade players also responded to the survey, they were excluded from this study because their schools had changed from elementary to junior high in the previous year, and their soccer-playing environment had largely changed at the half-year. All respondents provided informed consent by displaying and clicking on a screen before participating in the study. Respondents who agreed to participate in this survey answered the questionnaire voluntarily, and information was collected without revealing the identity of any individual participant to the researcher. The ethical review board of the authors’ institution approved the present study. This research was conducted based on the principles of the Declaration of Helsinki.

**Table 1:** Questionnaire regarding sports specialization and related factors in young male soccer players.

**Screening questionnaire (SC1–4)**
**SC1. Does your child currently play soccer in club teams?**
Yes/No/No children
**SC2. In what grade is your child who plays soccer?**
7th grade/8th grade/9th grade/Others
**SC3. What is the sex of your child as described in SC2?**
Male/Female
**SC4. How many days per week has your child played soccer (including practice and games) in the past 12 months?**
1 day per week/2 days per week/3 days per week/4 days per week/5 days per week/6 days per week/7 days per week/Others (less than 1 day per week)
**Main questionnaire (Q1–6)**
**Please answer regarding your child who plays soccer as described in the previous questionnaire (SC1–4)**
**Q1. How old is your child?**
years and months old
**Q2. At what age did your child start playing soccer, excluding recreational?**
years and months old
**Q3-1. Please select the sports your child participated in during seventh to ninth grade.**
Soccer/Swimming/Gymnastics/Track and field/Baseball/Softball/Volleyball/Basketball/Tennis/Soft tennis/Badminton/Table tennis/Kendo/Judo/Karate/Other () /No sports activity
**Q3-2. Please select the sports your child participated in during fourth to sixth grade.**
Soccer/Swimming/Gymnastics/Track and field/Baseball/Softball/Volleyball/Basketball/Tennis/Soft tennis/Badminton/Table tennis/Kendo/Judo/Karate/Other () /No sports activity
**Q4-1. Did your child consider soccer more important than other sports during seventh to ninth grade?**
Yes/No
**Q4-2. Did your child consider soccer more important than other sports during fourth to sixth grade?**
Yes/No
**Q5-1. How many months per year has your child played football (including practice and games) in the last 12 months during seventh to ninth grade?**
1 month/ 2 months/ 3 months/ 4 months/ 5 months/ 6 months/ 7 months/ 8 months/ 9 months/ 10 months/ 11 months/ 12 months
**Q5-2. How many months per year has your child played football (including practice and games) in the last 12 months during fourth to sixth grade?**
1 month/ 2 months/ 3 months/ 4 months/ 5 months/ 6 months/ 7 months/ 8 months/ 9 months/ 10 months/ 11 months/ 12 months
**Q6. Did your child quit other sports to focus on soccer?**
Yes, quit before third grade/Yes, quit during fourth to sixth grade/Yes, quit during seventh to ninth grade/No, continued other sports/No, plays only soccer

### Electronic questionnaire

The questionnaire consisted of four screening questions and six main questions (Table 1). The screening survey assessed whether the respondent had a child with the following criteria: (1) currently playing soccer in the club team, (2) in seventh to ninth grades, (3) male, and (4) playing soccer regularly for more than 1 day per week. If answers to the screening questions matched the inclusion criteria, the questionnaire continued to the next main survey. If the participant did not match even one of the inclusion criteria, the respondents were excluded from the survey. The main questions asked respondents about their sports activity (year, age at which they started playing soccer, participation in sports during seventh to ninth grades and during fourth to sixth grades), and status of specialization during the seventh to ninth grades and fourth to sixth grades. Status of sport specialization was determined with a widely used 3-point specialization scale [3, 4]. This scale was calculated from a series of three questions that asked (1) if the athlete quit other sports to focus on soccer; Q6, (2) if they viewed soccer as more important than other sports; Q4, and (3) if they trained or participated in soccer more than 8 months of the year; Q5 [14]. Specialization was classified as “high” for those who met all three criteria, “moderate” for those who met two, and “low” for those who met only zero to one. Those who answered “No, plays only soccer” to question 1 (Q6) were considered to meet this criterion.

### Statistical analysis

We summarized the data as means and standard deviations, frequencies and proportions (%) by specialization status. Using chi-square test, we compared the proportion of those who play only soccer by specialization status. Days of playing soccer per week and age starting soccer were compared by specialization status using one-way analysis of variance (ANOVA), along with Bonferroni correction performed as a post hoc test. All statistical analyses were performed using SPSS Statistics version 19.0 for Windows (IBM; Brush Prairie, WA, USA), and results were considered statistically significant with an α level of *P* < .05.

## Results

A total of 400 participants (mean age, 13.9 ± 0.9 years) completed the questionnaire. Table 2 presents the proportion of participants classified as high, moderate, and low specialization: 53.8% of participants demonstrated a high level of specialization, 32.3% demonstrated a moderate level of specialization, and 14.0% of participants demonstrated a low level of specialization. Among the participants, 74.5% considered soccer more important than other sports, 89.0% trained for more than 8 months of the year in soccer, and 74.0% had quit other sports to focus on soccer or playing only soccer. In total, 81.5% of participants and 92.1% of the high-specialization group trained 12 months of the year. Based on the results of chi-square test, the proportion of participants who playing only soccer was significantly higher in the high-specialization group (38.6%) than in the moderate-specialization (22.5%; *P* < .01) and low-specialization (7.1%; *P* < .01) groups, and it was significantly higher in the moderate- compared with the low-specialization group (*P* < .01). Based on the results of one-way ANOVA, the number of days spent playing soccer per week was significantly greater in the high-specialization group (4.6 ± 1.2 days, mean ± SD) than in the moderate- (4.1 ± 1.5 days; *P* < .01) and low-specialization (3.8 ± 1.7 days; *P* < .01) groups. Additionally, one-way ANOVA revealed that the age at which the participant started playing soccer was significantly earlier in the high-specialization group (7.8 ± 2.7 years) than in the moderate-specialization group (9.0 ± 2.9 years; *P* < .01). Regarding specialization status at grades 4 to 6 (9–12 years), 40.3% of participants demonstrated a high level of specialization, and 71.6% of the high-specialization group already demonstrated a high level of specialization.

**Table 2:** Frequency of sports participation and prevalence of injury.

	Specialization Status							
	Low		Moderate		High		Total	
Number	56	(14.0%)	129	(32.3%)	215	(53.8%)	400	(100.0%)
Soccer more important								
Yes	3	(5.4%)	80	(62.0%)	215	(100.0%)	298	(74.5%)
No	53	(94.6%)	49	(38.0%)	0	(0.0%)	102	(25.5%)
Months of participation								
1	8	(14.3%)	1	(0.8%)	0	(0.0%)	9	(2.3%)
2	0	(0.0%)	2	(1.6%)	0	(0.0%)	2	(0.5%)
3	3	(5.4%)	4	(3.1%)	0	(0.0%)	7	(1.8%)
4	4	(7.1%)	2	(1.6%)	0	(0.0%)	6	(1.5%)
5	1	(1.8%)	3	(2.3%)	0	(0.0%)	4	(1.0%)
6	7	(12.5%)	6	(4.7%)	0	(0.0%)	13	(3.3%)
7	1	(1.8%)	2	(1.6%)	0	(0.0%)	3	(0.8%)
8	1	(1.8%)	2	(1.6%)	4	(1.9%)	7	(1.8%)
9	1	(1.8%)	1	(0.8%)	3	(1.4%)	5	(1.3%)
10	1	(1.8%)	5	(3.9%)	6	(2.8%)	12	(3.0%)
11	0	(0.0%)	2	(1.6%)	4	(1.9%)	6	(1.5%)
12	29	(51.8%)	99	(76.7%)	198	(92.1%)	326	(81.5%)
Other sports								
Playing only soccer	**4**	**(7.1%)**	**29**	**(22.5%)** *	**83**	**(38.6%)** **	116	(29.0%)
Quit before grade 3 (–9 y)	2	(3.6%)	8	(6.2%)	36	(16.7%)	46	(11.5%)
Quit at grades 4–6 (9–12 y)	1	(1.8%)	24	(18.6%)	73	(34.0%)	98	(24.5%)
Quit after grade 7 (12 y–)	5	(8.9%)	8	(6.2%)	23	(10.7%)	36	(9.0%)
Continuing other sports	44	(78.6%)	60	(46.5%)	0	(0.0%)	104	(26.0%)
Days of playing soccer, mean (SD), d/wk	**3.8**	**(1.7)**	**4.1**	**(1.5)**	**4.6**	**(1.2)** **	4.3	(1.4)
Age starting soccer, mean (SD), y	8.8	(3.0)	9.0	(2.9)	**7.8**	**(2.7)** #	8.3	(2.8)
Specialization status at grades 4–6 (9–12 y)								
Other sports	16	(28.6%)	25	(19.4%)	21	(9.8%)	62	(15.5%)
Low	36	(64.3%)	15	(11.6%)	7	(3.3%)	58	(14.5%)
Moderate	4	(7.1%)	82	(63.6%)	33	(15.3%)	119	(29.8%)
High	0	(0.0%)	7	(5.4%)	154	(71.6%)	161	(40.3%)

Values in bold font indicate significantly different between groups.

*Significantly different from low level of specialization (*P* < .01).

**Significantly different from low and moderate level of specialization (*P* < .01).

#Significantly different from moderate level of specialization (*P* < .01).

## Discussion

The results of this study showed that 53.8% of young Japanese soccer players had a high specialization level, and 40.3% were already at a high level of specialization when they were in grades 4 to 6. Factors contributing to high specialization included year-round activity in soccer and rare participation (0%) in other sports. Additionally, 38.6% of athletes at the high specialization level reported no prior experience in sports other than soccer. These athletes were also active more days per week compared to those at moderate and low specialization levels. These results were consistent with our hypothesis. The level of specialization of young Japanese athletes has not been previously determined, and the results of this study may provide a baseline for comparing the sporting environment of young athletes with the findings of other studies.

The level of specialization of young Japanese soccer players in this study was higher than in previous research [5–7]. In addition, their level of specialization in grades 4–6 was similar to that reported in previous studies with older participants [5, 6]. Because high specialization is associated with the occurrence of overuse injuries [15], the high specialization of young Japanese soccer players potentially threatens their health status. In fact, the incidence of growth-related injuries among young Japanese soccer players [11, 16] is higher than that reported in a previous study from another country [17]. Osgood–Schlatter disease, which is most likely to be a problem in soccer players, tends to occur at the age of this study’s participants [18]. Soccer-related motions, including kicking, running, landing, and cutting, place a substantial load on knee extension, and the repetition of these loads is associated with a higher risk of developing Osgood–Schlatter disease [19]. To prevent these injuries, excessive training associated with early specialization must be avoided.

One reason for the high level of specialization was the number of months of activity per year. Although the criterion for specialization is ≥8 months of activity per year [3, 4], most of the participants in present study were active throughout the year. Sports activities for young players in Japan are typically conducted year-round rather than seasonally, and the present study clearly illustrates this situation. The number of months active per year was also related to the occurrence of overuse disorder [6], but it is not realistic to control the number of months active in a situation in which most players are active throughout the year. The results of the present study showed significant differences in the number of weekly activity days between the levels of specialization, which might be useful for controlling the amount of training.

Another reason for the high level of specialization was the low implementation of playing sports other than soccer. Only 26% of the participants continued to play other sports, which is much lower than the proportion reported in the previous study (77.9%) [7]. A previous study of German soccer players [20] found that they continued to play sports other than soccer until their late teenage years across all levels. Furthermore, approximately 30% of the participants had no experience in sports other than soccer, and 38.6% of those with a high level of specialization found themselves in a situation where they had no experience in sports other than soccer. On the other hand, according to a previous study of U.S. MLS professional soccer players [21], only 18.8% had no experience in any sport other than soccer. Meanwhile, the age at which participants started playing soccer was similar to that reported in a previous study [6] or later [20, 21]. This indicates that Japanese soccer players start playing soccer late and specialize early, often without significant experience in other sports. These trends align with those observed among non-U.S. players surveyed in a study of U.S. MLS professional soccer players [21]. In Japan, particularly among the U12 and U15 age groups, soccer enjoys immense popularity with numerous available teams [22], fostering a strong tendency toward early specialization in players’ athletic journeys. However, considering long-term athlete development [23], children are encouraged to participate in multiple sports, especially team ball sports, as early specialization in a single sport can increase the risk of overuse injuries in later youth stages [3, 10]. In Japan, it is crucial to create an environment where young soccer players can engage in multiple sports concurrently. Nonetheless, there is a concern in Japan that the overall training volume may be excessive for those participating in multiple sports [9], which needs careful management.

This study has several limitations. First, this was a cross-sectional study with a self-reported questionnaire in which parents recalled previous information. Thus, there is the possibility of recall bias in the results. However, we assumed that information on sports experience over the past several years can be collected satisfactorily. Second, the relationships between sports specialization and success or performance in the sports were not evaluated. Finally, we did not examine the relationship between sports specialization and the occurrence of injuries. The influence of sport specialization on injury occurrence and future injury risk in soccer players should be examined in the future. However, the result of the present study can provide a foundation for these future studies.

## Conclusion

We clarified the status of sports specialization and the relationships between specialization and training status in Japanese young soccer players. Japanese young soccer players have a tendency toward early sports specialization. Factors that contributed to the high specialization level were being active throughout the year and rarely continuing to play other sports. Training volume should be controlled for young soccer players of this age, and an environment that allows young players to engage in multiple sports simultaneously is needed to discourage early specialization.

## References

[1] Faude O, Kerper O, Multhaupt M, Winter C, Beziel K, Junge A, et al. Football to tackle overweight in children. Scand J Med Sci Sports. 2010;20 Suppl 1:103–10. doi: 10.1111/j.1600-0838.2009.01087.x . 20136766

[2] Bell DR, Post EG, Biese K, Bay C, Valovich McLeod T. Sport specialization and risk of overuse injuries: a systematic review with meta-analysis. Pediatrics. 2018;142(3):e20180657. doi: 10.1542/peds.2018-0657 . 30135085

[3] Jayanthi NA, Post EG, Laury TC, Fabricant PD. Health consequences of youth sport specialization. J Athl Train. 2019;54(10):1040–9. doi: 10.4085/1062-6050-380-18 ; PubMed Central PMCID: PMC6805065. 31633420 PMC6805065

[4] Jayanthi N, Pinkham C, Dugas L, Patrick B, Labella C. Sports specialization in young athletes: evidence-based recommendations. Sports Health. 2013;5(3):251–7. doi: 10.1177/1941738112464626 ; PubMed Central PMCID: PMC3658407. 24427397 PMC3658407

[5] Post EG, Biese KM, Schaefer DA, Watson AM, McGuine TA, Brooks MA, et al. Sport-specific associations of specialization and sex with overuse injury in youth athletes. Sports Health. 2020;12(1):36–42. doi: 10.1177/1941738119886855 ; PubMed Central PMCID: PMC6931179. 31724908 PMC6931179

[6] Post EG, Trigsted SM, Riekena JW, Hetzel S, McGuine TA, Brooks MA, et al. The association of sport specialization and training volume with injury history in youth athletes. Am J Sports Med. 2017;45(6):1405–12. doi: 10.1177/0363546517690848 . 28288281

[7] Bell DR, Post EG, Trigsted SM, Hetzel S, McGuine TA, Brooks MA. Prevalence of sport specialization in high school athletics: a 1-year observational study. Am J Sports Med. 2016;44(6):1469–74. doi: 10.1177/0363546516629943 . 26920433

[8] Shigematsu R, Katoh S, Suzuki K, Nakata Y, Sasai H. Sports specialization and sports-related injuries in Japanese school-aged children and adolescents: a retrospective descriptive study. Int J Environ Res Public Health. 2021;18(14):7369. doi: 10.3390/ijerph18147369 ; PubMed Central PMCID: PMC8307530. 34299823 PMC8307530

[9] Nagano Y, Oyama T. Association of sports sampling and training frequency with injury among school-age athletes in Japan. Phys Sportsmed. 2023;51(1):20–6. doi: 10.1080/00913847.2021.1973337 . 34433358

[10] Nagano Y, Oyama T. Early sport specialization trends and injuries in former high school athletes specialized in sports. Open Access J Sports Med. 2023;14:1–7. doi: 10.2147/OAJSM.S385554 ; PubMed Central PMCID: PMC9922066. 36785718 PMC9922066

[11] Sasaki S, Nagano Y, Koyama K. Factors associated with knee and heel pain in children: an observational web-based survey for 1,200 parents with young Japanese footballers aged in 8–12 years. European Journal of Sport Sciences. 2023;2(2):59–67. doi: 10.24018/ejsport.2023.2.2.75

[12] Sasaki S, Nagano Y. Observational Study of Growth-Related Knee Pain in Japanese Footballers Aged 12–15 Years: A Subsequent Series Following Children Aged 8–12 Years. European Journal of Sport Sciences. 2024;3(2):27–33. doi: 10.24018/ejsport.2024.3.2.155

[13] Hulley SB, Cummings SR, Browner WS, Grady DG, Newman TB. Designing Clinical Research. 4th ed. Philadelphia: Lippincott Williams & Wilkins; 2013.

[14] Post EG, Thein-Nissenbaum JM, Stiffler MR, Brooks MA, Bell DR, Sanfilippo JL, et al. High school sport specialization patterns of current division I athletes. Sports Health. 2017;9(2):148–53. doi: 10.1177/1941738116675455 ; PubMed Central PMCID: PMC5349389. 27807260 PMC5349389

[15] Bell DR, Post EG, Trigsted SM, Schaefer DA, McGuine TA, Watson AM, et al. Sport specialization characteristics between rural and suburban high school athletes. Orthop J Sports Med. 2018;6(1):2325967117751386. doi: 10.1177/2325967117751386 ; PubMed Central PMCID: PMC5777568. 29376086 PMC5777568

[16] Iwame T, Matsuura T, Suzue N, Iwase J, Uemura H, Sairyo K. Factors Associated With Knee Pain and Heel Pain in Youth Soccer Players Aged 8 to 12 Years. Orthop J Sports Med. 2019;7(11):2325967119883370. doi: 10.1177/2325967119883370 ; PubMed Central PMCID: PMC6868579. 31799330 PMC6868579

[17] Materne O, Chamari K, Farooq A, Weir A, Holmich P, Bahr R, et al. Injury incidence and burden in a youth elite football academy: a four-season prospective study of 551 players aged from under 9 to under 19 years. Br J Sports Med. 2021;55(9):493–500. doi: 10.1136/bjsports-2020-102859 . 33199359

[18] Materne O, Chamari K, Farooq A, Tabben M, Weir A, Holmich P, et al. Shedding light on incidence and burden of physeal injuries in a youth elite football academy: A 4-season prospective study. Scand J Med Sci Sports. 2022;32(1):165–76. doi: 10.1111/sms.14059 . 34551163

[19] Itoh G, Ishii H, Kato H, Nagano Y, Hayashi H, Funasaki H. Risk assessment of the onset of Osgood-Schlatter disease using kinetic analysis of various motions in sports. PLoS One. 2018;13(1):e0190503. Epub 2018/01/09. doi: 10.1371/journal.pone.0190503 ; PubMed Central PMCID: PMC5757930. 29309422 PMC5757930

[20] Hornig M, Aust F, Gullich A. Practice and play in the development of German top-level professional football players. Eur J Sport Sci. 2016;16(1):96–105. doi: 10.1080/17461391.2014.982204 . 25440296

[21] Knapik DM, Rizzone KH, Voos JE. Timing and Reasons Behind Single-Sport Specialization in Soccer: A Survey of 64 Major League Soccer Athletes. Sports Health. 2020:1941738120911373. doi: 10.1177/1941738120911373 . 32286914 PMC7787568

[22] Association JF. Data box 2022 [cited 2023 10/14]. Available from: https://www.jfa.jp/eng/about_jfa/organization/databox/team.html.

[23] Balyi I, Way R, Higgs C. Long-term athlete development: Human Kinetics; 2013.

